# Nanoparticle and Gelation Stabilized Functional Composites of an Ionic Salt in a Hydrophobic Polymer Matrix

**DOI:** 10.1371/journal.pone.0088125

**Published:** 2014-02-06

**Authors:** Selin Kanyas, Derya Aydın, Riza Kizilel, A. Levent Demirel, Seda Kizilel

**Affiliations:** 1 Material Science and Engineering, Koc University, Sariyer, Istanbul, Turkey; 2 Department of Chemical and Biological Engineering, Koc University, Sariyer, Istanbul, Turkey; 3 Koc University-TUPRAS Energy Center (KUTEM), Koc University, Sariyer, Istanbul, Turkey; 4 Department of Chemistry, Koc University, Sariyer, Istanbul, Turkey; University of Kansas, United States of America

## Abstract

Polymer composites consisted of small hydrophilic pockets homogeneously dispersed in a hydrophobic polymer matrix are important in many applications where controlled release of the functional agent from the hydrophilic phase is needed. As an example, a release of biomolecules or drugs from therapeutic formulations or release of salt in anti-icing application can be mentioned. Here, we report a method for preparation of such a composite material consisted of small KCOOH salt pockets distributed in the styrene-butadiene-styrene (SBS) polymer matrix and demonstrate its effectiveness in anti-icing coatings. The mixtures of the aqueous KCOOH and SBS-cyclohexane solutions were firstly stabilized by adding silica nanoparticles to the emulsions and, even more, by gelation of the aqueous phase by agarose. The emulsions were observed in optical microscope to check its stability in time and characterized by rheological measurements. The dry composite materials were obtained via casting the emulsions onto the glass substrates and evaporations of the organic solvent. Composite polymer films were characterized by water contact angle (WCA) measurements. The release of KCOOH salt into water and the freezing delay experiments of water droplets on dry composite films demonstrated their anti-icing properties. It has been concluded that hydrophobic and thermoplastic SBS polymer allows incorporation of the hydrophilic pockets/phases through our technique that opens the possibility for controlled delivering of anti-icing agents from the composite.

## Introduction

Incorporation of hydrophilic functional agents within a hydrophobic polymer matrix has been a conventional challenge in materials science. This challenge can be addressed by forming stable liquid emulsions and using them as templates to form solid-like composites. [1]–[17] Templates of emulsions have been used for the two main approaches. The first of these approaches yields porous monolith materials. [2], [4], [10], [11] This approach requires transformation of the continuous phase of an emulsion into a solid matrix. Subsequently, dispersed droplets are removed by evaporating or dissolving in a suitable liquid, leaving spherical pores behind. [10], [11] Such porous monoliths are attractive potential materials for filtering aerosols. Imhof and Pine have templated micro-emulsions of droplet size ranging from 100 to 200 nm. [10] They have obtained a porous monolith by removal of oil droplets within aqueous continuous medium and gelation of the aqueous medium by sol-gel method. [11] In a previous study, Binks prepared a porous silica monolith by silica particles alone. [4] Imhof and Pine have employed sol-gel processing to cure the continuous phase. [10] Such porous monoliths are also proposed to be used as adsorbents, catalytic supports, light-weight structural materials, insulators besides their potential application as filters. [4], [10], [11], [14] The second approach involves precipitation or coating of a target material around the droplets in order to form core-shell structure. [1], [6], [16] The resultant capsules are subsequently isolated from the continuous phase and dried. Wei and Wan investigated the importance of self-assembled layers at the interface to form stable capsules. [16] The authors used polyanilin coated hollow microspheres for self-assembly of anilin monomers around oil-in-water (o/w) droplets in emulsion, and polymerized the shell subsequently. Such capsules are promising to be used as delivery vehicles for controlled-release encapsulation, drug delivery, protection of biologically active agents [6], [16].

In general, these two approaches of emulsion templating method have been considered for different applications. Macroporous systems are proposed to serve mostly as filtering systems, whereas the capsules have been considered to encapsulate either nanoparticles [18] or biomaterials such as drugs, food and cosmetics. [1], [6], [16], [17] In this study, we combined the two approaches through particle stabilization to design a functional polymer composite that includes an anti-icing agent. We used nanoparticles to stabilize the emulsion which is also known as Pickering emulsions method. It is well established that, as an alternative to surface active molecules, small solid particles attach at fluid/fluid interfaces of two immiscible mediums when the particles are partially wettable by both mediums. [19]–[21] Attachment of Pickering particles at the interface is thermodynamically favorable phenomenon. Their attachment to the interface is principally irreversible. [22], [23] The irreversibility of the attachment is a striking difference and an advantage relative to stabilization with conventional surfactant molecules. The very high energy of attachment of particles to interfaces yields remarkable stability against coalescence which surfactant molecules do not perform. [4] Unlike Pickering particles, surfactant molecules are held at the interface by dynamic equilibrium; they rapidly adsorb and desorb at the interface, which makes their stabilization weaker [24].

We designed a composite membrane coating, which integrates a hydrophobic polymer adhesive base and hydrophilic aqueous phase that incorporates anti-icing agent. Ionic salts are widely used agents for this purpose. The functional membrane consists of a base which is non-polar SBS block copolymer, and compatible with most non-polar surfaces. The hydrophilic domains include spherical droplets that store and induce controlled release of the anti-icing agent potassium formate salt (KCOOH). In order to avoid deformed shapes, enhance restricted permeability, and reinforce rheological properties, we included functional domains in agarose gel within the designed material. We used Pickering emulsion droplets as templates for the functional domains, [25], [26] which encapsulate anti-icing KCOOH in the functional membrane coating. KCOOH has been preferred as an anti-icing agent over common salts, such as CaCl_2_, NaCl, or MgCl_2_, due to its higher freezing point depression effect, low corrosion and, environmentally safe properties. [27]–[30] The advantages of this material prepared in this study are the restricted permeability of the composite structure towards the functional agent, and stability of the template during drying process.

Here, we report the design of a functional polymer composite consisting of KCOOH salt pockets dissolved in hydrophilic gel or aqueous medium, where this phase was dispersed in SBS polymer matrix. We demonstrate the effectiveness of this polymer composite as anti-icing coatings, which could be utilized for various applications. In order to achieve this goal, hydrogel filled and nanoparticle stabilized emulsions based on SBS that contain about 9–25 (v/v) % internal phase and exhibit significantly enhanced mechanical properties are investigated. The effects of experimental design parameters on the morphology of wet and dry emulsions, mechanical properties of wet emulsions, degree of hydrophobicity of templated dry emulsions, salt release and anti-icing properties of surface templated dry membranes are described and discussed in detail. The main thrust of this study is the unique functionality of SBS based emulsions with anti-icing agents, and possibility of releasing this water soluble agent into the surrounding medium to attain anti-icing property. Different from other previous studies where oil or hydrophobic solvent have been used, a hydrophobic block copolymer (SBS) has been used here to form the continuous phase of nanoparticle stabilized emulsions. In addition, both continuous and dispersed phases have been retained upon templating of the emulsion on surfaces in order to make use of desirable properties of both phases. One of the advantages of retaining continuous phase of this emulsion is the possibility of incorporation of this emulsion into other hydrophobic mediums, enhancing mechanical strength and durability. Furthermore, the presence of aqueous phase and delivery agent in this stable emulsion will result in functionalized hydrophobic medium that would otherwise be incompatible with the delivery material. The silica nanoparticles used here not only potentially stabilize the emulsions but also introduce additional benefits such as improved mechanical property, and allows for controlled release of KCOOH through the internal phase domains and through the particle shells. To the best of our knowledge, this is the first report of the preparation of a surface coating polymer membrane formed by the combined system of Pickering emulsion and solvent evaporation. This method is expected to be a potential candidate for the design and synthesis of functional surface coating polymeric membranes.

## Materials and Methods

### Materials

Styrene-butadiene-styrene block copolymer (SBS, S:B wt fraction: 70∶30) and Potassium formate (KCOOH) salt (99%) and Sodium dodecyl sulfate (SDS) was purchased from Sigma in powder form. Agarose powder was purchased from Sigma-Aldrich. Silica nanoparticles (AEROSIL 816 ®) with particle diameter of 12 nm were kindly supplied by Degussa in powder form. Cyclohexane (99.9%), acetone (99%), ethanol (99%) were purchased from Merck. Glass Petri-dishes and glass microscope slides were purchased from Nunc. Deionized water with an electrical resistance of 17 MΩcm was used in our experiments (Purelab Option, ELGA).

### Preparation of Emulsions using SDS as Surfactant

Emulsions were prepared with SDS as surfactant to investigate the stabilization potential. KCOOH (0.05 gr/mL) was dissolved in distilled water (dH_2_O) to prepare the internal phase of the emulsions. SDS solution was added into the aqueous phase along with KCOOH with a concentration of 0.016 g/ml. Next, internal phase was emulsified in 1 ml SBS polymer in cyclohexane (110 mg/ml).

### Preparation of Silica Nanoparticle and SBS Solutions

Functional silica nanoparticles were used to stabilize emulsions. Silica AEROSIL 816 ® nanoparticles were dispersed in cyclohexane with three different concentrations: 0.4, 0.7, and 1.0 gram in 100 mL cyclohexane. The dispersion was carried out by high energy application via ultra-sonication tip (73 MS, 50% amplitude, 50 Hz frequency) for 3 minutes. Ice bath was applied every 45 seconds to avoid excessive temperature rises. During the period between nanoparticle dispersion and emulsion preparation, the nanoparticle suspension is kept in ultrasonic bath in order to avoid agglomerations that may arise from steady positioning. Dynamic Light Scattering (DLS) measurements indicated that agglomeration could not be fully avoided. Nanoparticle dispersions of all concentrations resulted in particle sizes within the range of 100–150 nm. The SBS stock solution is prepared by mixing 110 mg/ml SBS in cyclohexane. The mixture is stirred on magnetic stirrer at room temperature.

### Preparation of the Emulsion without Gelation of the Internal Phase

The moisture in the solid KCOOH was eliminated through freeze-drying. An aqueous KCOOH stock solution (0.5 g/mL) was prepared by dissolving the salt in distilled water at room temperature. In order to obtain emulsion templates with just aqueous KCOOH solution in the dispersed phase, 0.5 mL of nanoparticle stock solution was added into aqueous KCOOH solution in the amounts of 0.5, 0.25, or 0.15 mL to obtain emulsions with 9, 14, or 25% (v/v) internal phase fractions, respectively. To this solution 1 mL of SBS stock solution is added to obtain emulsions with SBS in the continuous phase. The stability of the emulsions in vials was observed for 3 days at room temperature. For parametric sensitivity experiments, emulsion solutions containing altered concentrations of nanoparticles and internal phase volume fraction were examined. Table 1 demonstrates the concentrations for each parameter used to investigate the properties of emulsions.

**Table 1:** Compositions of the emulsions prepared to investigate the effects on the properties of emulsion templates.^a^

Oil	Internal Volume Fraction % (v/v)	Silica nanoparticle concentration instock solution % (w/v)
SBS Solution	**9**	**0.4**
SBS Solution	**14**	**0.7**
SBS Solution	**25**	**1.0**

a Internal phase volume fraction was calculated with respect to the emulsion total volume (water+particles+SBS solution).

### Preparation of the Emulsion with Gelation of the Internal Phase

Internal phase of the emulsions were prepared with agarose to investigate the influence of gel medium on the stability of the emulsions and on the release properties of the anti-icing agent KCOOH. Agarose solution was prepared by adding 1% (w/w) agar powder in distilled water. The mixture was cooked in microwave oven until it boiled and all powder was dissolved. Subsequently 0.5 g of KCOOH was dissolved in 1 mL of agarose solution, which yielded 0.5 g/ml KCOOH (s) salt solution in agarose. Three different fractions of internal volume of salty agar solution were added into three different concentrations of nanoparticle-cyclohexane dispersion. The particle dispersion was kept at room temperature. Stock nanoparticle solution concentrations of 0.4% (w/v), 0.7% (w/v), 1.0% (w/v), and internal volume fractions of 9% (v/v), 14% (v/v), 25% (v/v) were prepared (Table S1). For example, for an internal volume fraction of 25% (v/v), 0.5 mL of the KCOOH agar solution was added into 0.5 mL of nanoparticle stock solution phase by vortex at maximum speed for 5 seconds. As hot agarose solution was mixed in the cyclohexane, the aqueous solution formed well-dispersed droplets which were stabilized by nanoparticles. The stabilized agarose droplets instantly cooled as they mixed with the nanoparticle solution at room temperature. As agarose solution turned into gel phase, hydrophilic gel droplets that were dispersed in cyclohexane formed an emulsion. Subsequently, 1 ml of the concentrated SBS polymer in cyclohexane (110 mg/ml) was added drop-wise into 1 mL of the KCOOH, agar, and nanoparticle solution with gentle mixing.

### Casting of Emulsions on Substrate Surfaces

Prepared emulsions were casted on glass substrates to further investigate the morphology and anti-icing property of the composite structure. Microscope glass slides were rinsed with ethanol and distilled water. The substrates were dried with nitrogen and 100 µl of each emulsion solution was deposited on glass slides separately. The cast membranes on glass slides were dried at 15°C. The membranes were considered to be dry when the solvent evaporated from both phases of the emulsion, and membranes appeared elastic.

### Surface Characterization

*Optical Microscopy, Scanning Electron Microscopy (SEM).* Changes in the final microstructures of the emulsion samples were characterized through optical microscopy. Emulsion samples were placed on glass slides separately, and were observed under microscope (Nikon, Eclipse Ni-U) in both wet and dry states. Optical micrographs were obtained by placing emulsion samples on previously cut square shaped glass slides. They were then placed in an optical microscope attached to a DS camera control unit (Model: DS-L3) both before (wet) or after solvent evaporation (dry). The surface images were imported to a software program (Kameram) for analysis of the droplet sizes and morphologies. Morphologies of both wet and dry castings were found to be dependent on the constituents of the dispersed phase. The effects of particle concentration in the emulsion, internal phase volume fraction, and gelation of the internal phase on the morphology of membranes have been studied (Table 1). The morphology of an emulsion sample that contains agarose gel in the internal phase was analyzed via SEM (Zeiss Ultra Plus, Bruker; Optronik Ltd Sti, Ankara, Turkey).

*Contact Angle Measurements.* The surface hydrophobicity of composite coatings was investigated by contact angle measurements. Water contact angles (WCA) on surfaces of dry cast membranes were measured at room temperature and ambient relative humidity using goniometer (DataPhysics Instruments, Germany). The membrane surfaces were 0.5–1 cm^2^ wide. The membranes were characterized after solvent evaporation. A water droplet of 5 µl was deposited on the surface of each dry membrane and advancing contact angles were measured. All of the values reported are the average of four measurements taken at different locations of an individual membrane and have a maximum error of ±4°.

### Rheological Characterization

Rheological behavior of emulsions prepared with altered experimental conditions were characterized at 25°C using a stress-strain controlling rheometer. (Discovery Hybrid Series-2, Thermal Analysis (TA)) Experiments were performed with a profiled parallel-plate geometry, with 20 mm plate diameter, and with a mechanically set gap of 950 µm. Flow tests were conducted to determine viscosities of the wet emulsions by gradually increasing the shear rate from 0.01 to 100 1/s. Oscillatory tests were conducted to measure G’and G’’ (storage and loss moduli) of the wet emulsions by frequency sweep from 1 to 100 Hz with a strain set at 0.2%.

### Salt Release Characterization

The release of KCOOH from dry composite membranes was investigated by immersing samples in water and the amount of salt release was measured at specific time points using Ion Selective Electrode (WTW Potassium Combination Electrode K 800). First, wet emulsions were poured into petri dishes and dried for 24 hours in order to obtain dry templates. Next, dry composite membranes were incubated in 20 ml deionized water for different time periods without changing the incubation water medium. The amount of KCOOH released was calculated using measured K+ concentrations for each dry composite membrane.

### Anti-icing Property Characterization

The anti-icing property of functional composite membranes was investigated by observation of freezing rate of water droplet on membrane surface. Freezing of water droplets on composite materials was monitored by using temperature and humidity controlled chamber (Teknofil Co.). Dry membranes were prepared on glass slides. Next, about 100 µL of water were dropped onto each membrane surface and incubated in the chamber at −14°C plate, 5°C ambient temperature, and ∼50% humidity conditions. Duration of the freezing of water droplets were recorded based on the disappearance of transparency of water droplets. In order to study anti-icing property of membranes upon repeated water exposures, subsequent incubation of surfaces in water were applied and salt release was measured after each incubation. The procedure was repeated for four times.

## Results and Discussion

**Figure 1:**
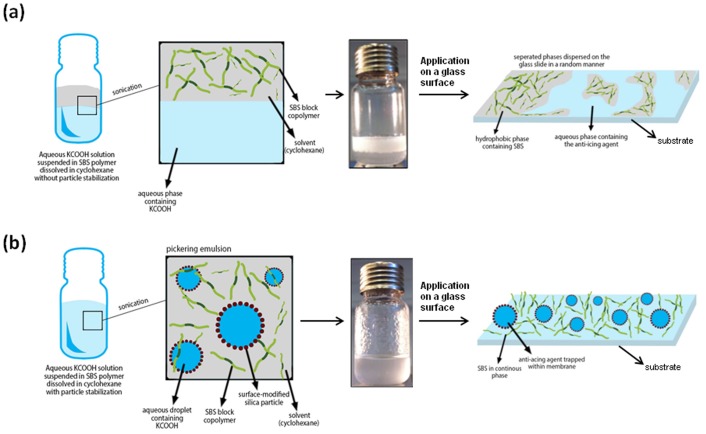
Schematic representation of the steps for casting of solutions prepared with aqueous KCOOH solution suspended in SBS polymer (a) without particle stabilization, or (b) with particle stabilization.

**Figure 2:**
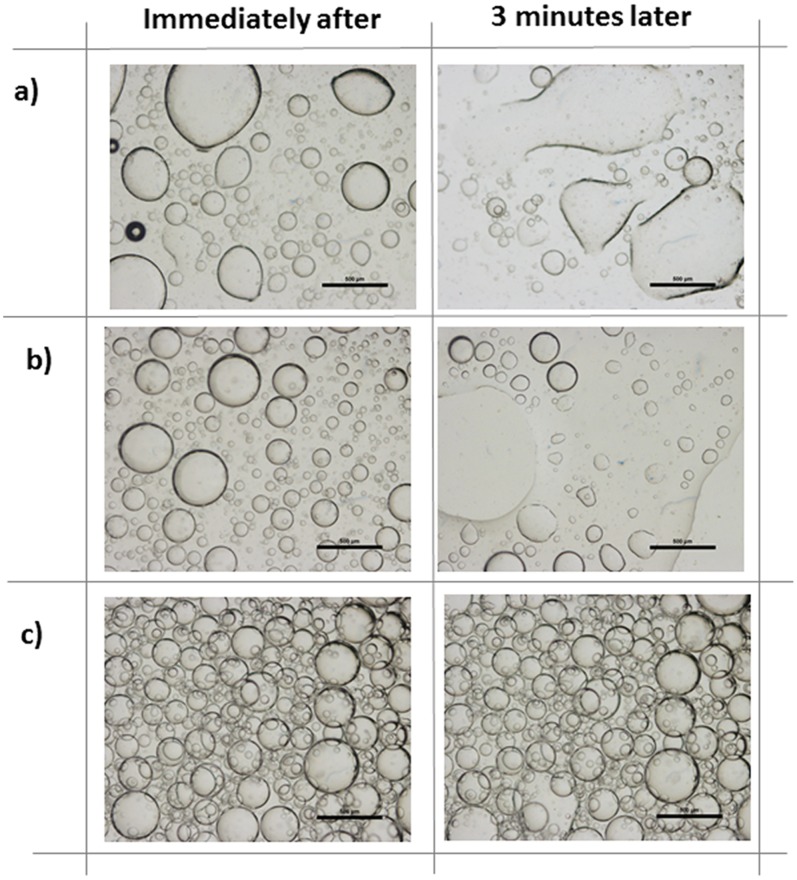
Optical microscope images of wet emulsions with internal volume fraction φ = 25% (v/v) prepared by different stabilization methods for immediately after and 3 minutes after preparation of emulsions. (a) Wet emulsions without stabilizing agent, (b) wet emulsions prepared with SDS surfactant as the stabilizing agent, (c) wet emulsions prepared by nanoparticle stabilization. Scale bar is 500 µm.

Stable emulsions of aqueous KCOOH solutions were prepared in SBS/cyclohexane solution using semi-hydrophobic silica nanoparticles (Figure 1). As shown in Figure 2a, without silica nanoparticles the emulsion was not stable and separated into two phases. Addition of semi-hydrophobic nanoparticles resulted in homogenous emulsions which were stable up to three months. Semi-hydrophobic silica nanoparticles have tendency to locate at the aqueous/organic interface and minimize the interfacial energy of the system. The amount of nanoparticle used is critical both for the long-term stability of the emulsion and for obtaining a homogenously dispersed salt pockets in the hydrophobic SBS matrix in the dry state.

In order to investigate the stabilization potency of different mechanisms, emulsions prepared with different methods were compared. First, emulsions were prepared without any additional stabilization attempt, then they were prepared by a long tailed ionic surfactant SDS and finally surface modified hydrophobic silica nanoparticles (AEROSIL 816®) have been used. Since the main goal was to develop an ideal stabilization technique for the proposed material mixture, initially no gel was introduced into the system. Droplet formation upon shear and phase separation have been observed in micro-scale under optical microscope immediately and 3 minutes after preparation. (Figure 2). The results show that, at the very instant of shear exposure, all emulsions yield to spherically shaped internal phase aqueous KCOOH droplets suspended in the continuous SBS copolymer solution medium. When stabilization agent is not used however, many of the aqueous droplets merge with time. Three minutes after deposition on the surface, unstable internal phase droplets were distorted and swiftly merged upon slightest collusion and yield phase separation (Figure 2a). Next, surfactant material was used to test the effect on emulsion stabilization. SDS was added into the aqueous phase along with KCOOH, before the internal phase was emulsified with the hydrophobic polymeric phase. Introduction of SDS into the system yielded stabilization of wet emulsions to some extent. Droplets with irregular sizes were formed, where many of them were not durable enough to survive beyond 3 minutes. (Figure 2b). As a final approach, particles are introduced into the continuous phase prior to emulsification in order to obtain robust and stable emulsions in this study. Unlike conventional surfactant molecules, functional nanoparticles were adequate to maintain well dispersed spherical droplets of the internal aqueous phase (Figure 2c). This demonstrated the superiority of the particle stabilization technique over conventional surfactants in our system. Particle self assembly around the droplets has the advantages of being irreversible and energetically more favorable over assembly of surfactant molecules.

**Figure 3:**
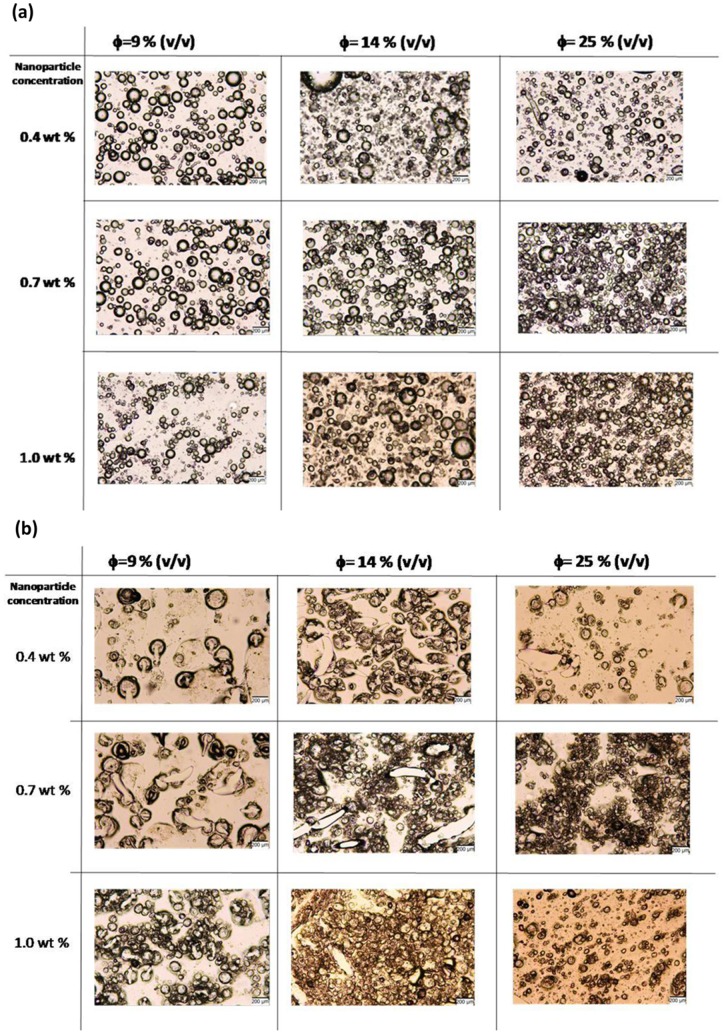
Optical microscope images of emulsions for altered concentrations of nanoparticles and internal phase volume fractions. Internal phase does not contain agarose gel. (a) Wet and, (b) dry emulsions. Scale bar represents 200 µm.

Figure 3 shows optical microscope images of emulsions and composite dry films on glass substrates at different nanoparticle weight percentages and at different volume fractions of the aqueous salt solutions. As volume fraction of the aqueous phase increased, more nanoparticles were needed to stabilize the emulsion (Figure 3a). When these emulsions were coated on glass substrates and dried, well-dispersed salt pockets were observed in the films with some irregular shapes and smaller sizes due to water evaporation and shrinkage (Figure 3b).

**Figure 4:**
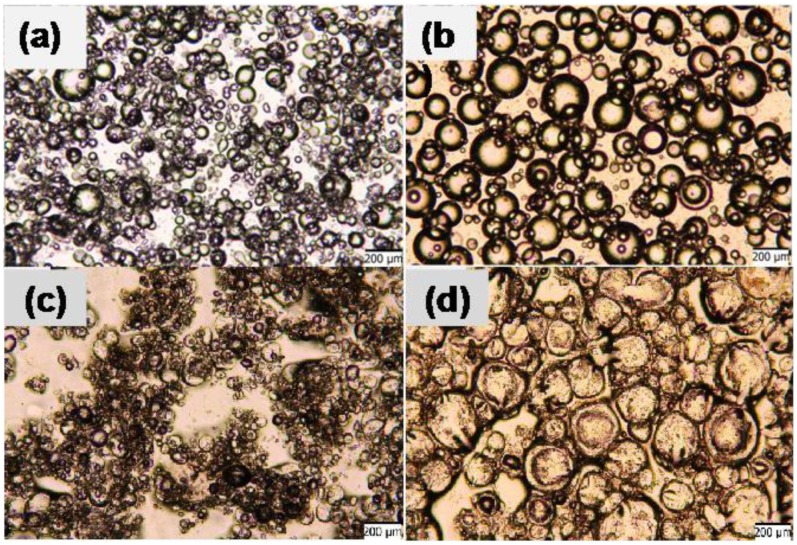
Optical microscope images of wet and dry emulsions prepared with internal volume fraction Φ = 0.33, and nanoparticle concentration of 0.7% (w/w). (a) Wet emulsions prepared without agar gel in the dispersed phase, (b) wet emulsions prepared with agar gel in the dispersed phase, (c) dry emulsions prepared without agar gel in the dispersed phase, (d) dry emulsions prepared with agar gel in the dispersed phase. Scale bar is 200 µm.

To maintain better dispersion and well-defined salt pockets in the dry state, we have taken advantage of agar gel to encapsulate the nanoparticle stabilized aqueous salt solution droplets. In Figure 4, the dispersions of aqueous/salt droplets in the emulsion and in the dry state are compared with those having agar gels in the aqueous phase. The droplets having agar gels in the aqueous phase were larger compared to those without gel (Figure 4a, 3b). More importantly, in the dry state droplets with gel maintained their well-defined spherical shapes with slight distortions due to shrinkage during drying. The gel and nanoparticle stabilized salt pockets were also much more homogenously dispersed in the dry films compared to those stabilized only by nanoparticles (Figure 4c, 3d). Furthermore, the salt pockets having gels in the aqueous phase were resistant to being cracked by capillary effects during evaporation of the solvents. The elimination of cracks allows better encapsulation of the salt in the pockets and its controlled release when needed. Better controlled release profile of the functional agent (salt in this case) from the gel domains to the surrounding is important in applications such as preventing ice formation by salt release.

**Figure 5:**
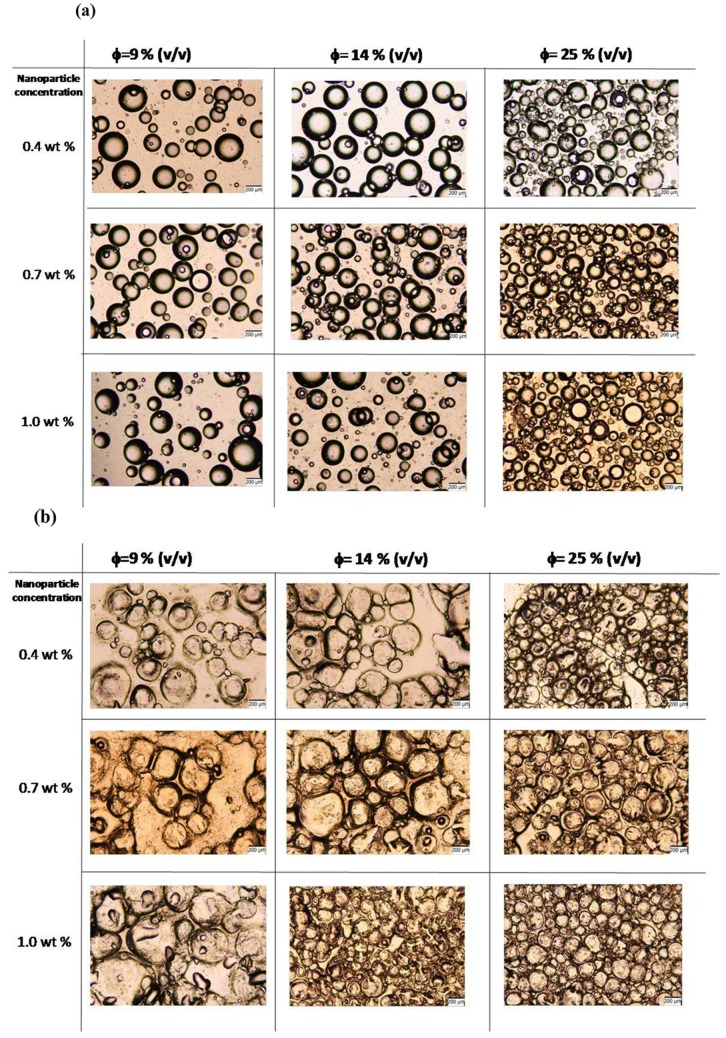
Optical microscope images of emulsions for altered concentrations of nanoparticles and internal phase volume fractions. Internal phase contains agarose gel. (a)Wet and, (b) dry emulsions. Scale bar represents 200 µm.

Figure 5 shows the effect of nanoparticle weight percentages and volume fraction of the aqueous phase on the homogeneity of droplets in emulsion and in dry state for emulsions prepared with agarose gel in the aqueous phase. The droplets of aqueous phase in emulsion and salt pockets in the dry state were larger in size compared to those stabilized only by nanoparticles. In the presence of agar gel, the size of the emulsion droplets and their homogeneity were dependent more on the volume fraction of the aqueous phase. Figure S1a displays the distribution of droplet size for wet emulsions formed with 17% (v/v) internal volume fraction and 1.0% (w/v) nanoparticle concentration. Gel domains showed a wide range of sizes from 20 µm to 300 µm in diameter. The droplets without gel had narrower size distribution between 20–180 µm. Figure 5a shows that low internal phase volume fraction (9% (v/v)) and low nanoparticle concentration lead to the formation of large (200–400 µm) functional domains (Figure 5a). Nanoparticles in the system tend to minimize the interfacial area between the continuous polymer phase and the aqueous gel phase. However, full coverage requires reduction in the surface area of functional droplets, thus increase in their size. The supportive gel structure contributes to the enlargement of emulsion droplets. As particle concentration increased, greater interfacial area, accompanied by smaller droplet size and greater number of droplets per unit area were obtained (Figure 5). This effect of particle concentration on droplet size has also been observed on the templated dry membranes (Figure 5b). It should also be noted that increasing internal volume phase fraction resulted in an increased contact of droplets in wet state and dry state. When internal phase volume fraction was high, continuous phase SBS polymer base was completely occupied by smaller functional domains, which were slightly deformed upon drying due to pressing on each other (Figure 5b).

These observations related to the decrease in droplet size with an increase in internal phase volume fraction or increases in nanoparticle solution concentration are also consistent with the previous findings of water-in-oil emulsion studies. For example, Akartuna et al. found that increasing the concentration of poly(vinylidene difluoride) (PVDF) particles lead to a decrease in droplet size of wet emulsions prepared with water-in-oil emulsions. [2] This was due to the higher viscosity of the original nanoparticle solution, which caused higher shear stress on the aqueous phase droplets during mixing. [31], [32] Similarly, increasing water content in the emulsions at a fixed particle concentration decreased droplet size. The reason for this effect was due to increased viscosity of the emulsion with high water content, which resulted in random close packing for the droplets [33].

The emulsions prepared in this study with agarose gel in the internal phase, have defined spherical shapes for dispersed droplets. Gelation of the internal phase resulted in the formation of highly dense and stiff droplets within the emulsions, which influenced both the size and the shape of the functional domains. The intended function of nanoparticles for the emulsion templates prepared in this study has primarily been the stabilization of the emulsions for a long period of time such that homogeneously distributed functional domains in the final membrane could be obtained. The stabilization effect of particles was better distinguished in the samples where agarose gel was not present in the internal phase, since gel cores remained stable at all nanoparticle concentrations used. In the samples prepared with agarose gel, the gel domains do not sustain the droplets’ perfect spherical shapes in dry state; however, they remained stiff enough to maintain a three dimensional closed shape (Figure 5). In addition to the contribution to stability of emulsions, nanoparticles have effects on gel droplet size and on the bridging behavior between stable droplets. Dry domains were slightly smaller but comparable in size to their initial wet droplet phase.

**Figure 6:**
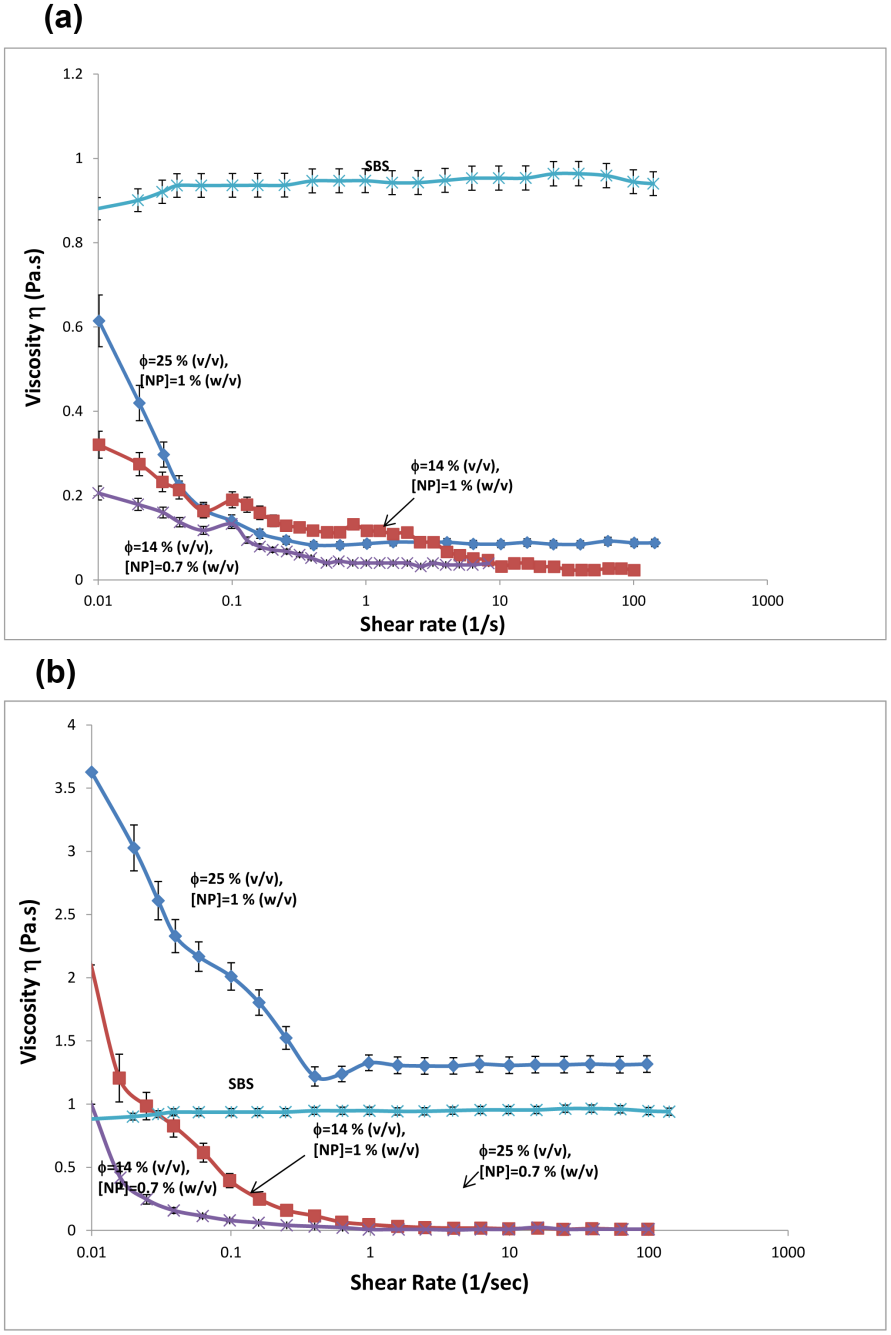
Viscosity versus shear rate profiles for emulsions with (a) no gelation of the internal phase, (b) gelation of the internal phase. Viscosity versus shear rate profile for homogeneous SBS solution has been included for comparison. Data indicates the average of at least three observations with corresponding standard deviations.

Figure 6 shows the viscosity of the emulsions as a function of shear rate in comparison to that of SBS solution. SBS solution in cyclohexane showed nearly constant viscosity of ∼0.95 Pa.s throughout the shear rate range of ∼0.01–100 Hz at room temperature. The observation of Newtonian behavior indicates that a maximum shear rate of 100 Hz is not large enough to break polymer entanglements. In other words, the slowest polymer relaxation process in the solution is faster than the maximum shear rate applied. The addition of the aqueous phase together with the emulsion stabilizing nanoparticles into the polymer solution decreased the viscosity compared to that of the polymer solution, but the emulsions showed shear thinning behavior (Figure 6a). The major reason of the overall decrease in viscosity is due to much lower viscosity of the aqueous phase compared to the polymer solution. The comparison of the emulsion viscosities at different aqueous phase volume fraction Φ and different nanoparticle percentages showed that the emulsion viscosity decreases both with decreasing Φ and with decreasing amount of nanoparticles. This observation together with the shear thinning behavior suggests that the interaction of the nanoparticle stabilized aqueous domains with each other contributes significantly to the viscosity of the emulsions. Adding more nanoparticles increases the stability of the aqueous domains and enhances the viscosity which is consistent with the previous studies. [32] In addition, more nanoparticles can stabilize more interfaces. Therefore, with increasing amount of nanoparticles, the size of aqueous domains decreases and the number of domains increases. Smaller aqueous domains result in larger interfacial area and enhanced interactions which lead to enhanced viscosity at fixed shear rate. [32] Similarly, increasing volume fraction of aqueous phase also increases the number of the aqueous domains and increases the viscosity of the emulsion. The addition of the aqueous phase into the polymer solution causes structuring in the emulsion at zero-shear. The structure is disrupted even at the slowest shear rate indicating rather slow dynamics in the emulsions. The breakdown of the structure with increasing shear rate decreases the viscosity further and leads to the shear thinning behavior.

When the aqueous phase contained agarose and formed gel, the viscosity of the emulsions was larger compared to that of SBS solution at zero shear rate (Figure 6b). The emulsions showed significant shear thinning behavior and the viscosity decreased sharply below the viscosity of SBS solution, except for emulsion containing 25 volume % gelled aqueous phase. Shear thinning in emulsions has commonly been observed and attributed to the disruption of the aqueous domains under high shear. [34], [35] Similar to the emulsions without agarose, viscosity of the emulsions with agarose in the aqueous phase also increased with both the volume fraction of the aqueous phase and the amount of nanoparticles. This indicates increased stability, increased number of aqueous domains and decreased domain size.

Storage modulus (G’) and loss modulus (G”) of emulsions with or without gelation in the aqueous phase showed a transition from predominantly viscous behavior (G”>G’) at low oscillation frequencies to elastic behavior (G’>G”) at higher frequencies (Fig. S2). This transition happened at ∼40 Hz for emulsions without gelled aqueous phase and at ∼2 Hz with gelled aqueous phase. This is consistent with the larger viscosity and slower dynamics in emulsions having gelled aqueous phase.

**Figure 7:**
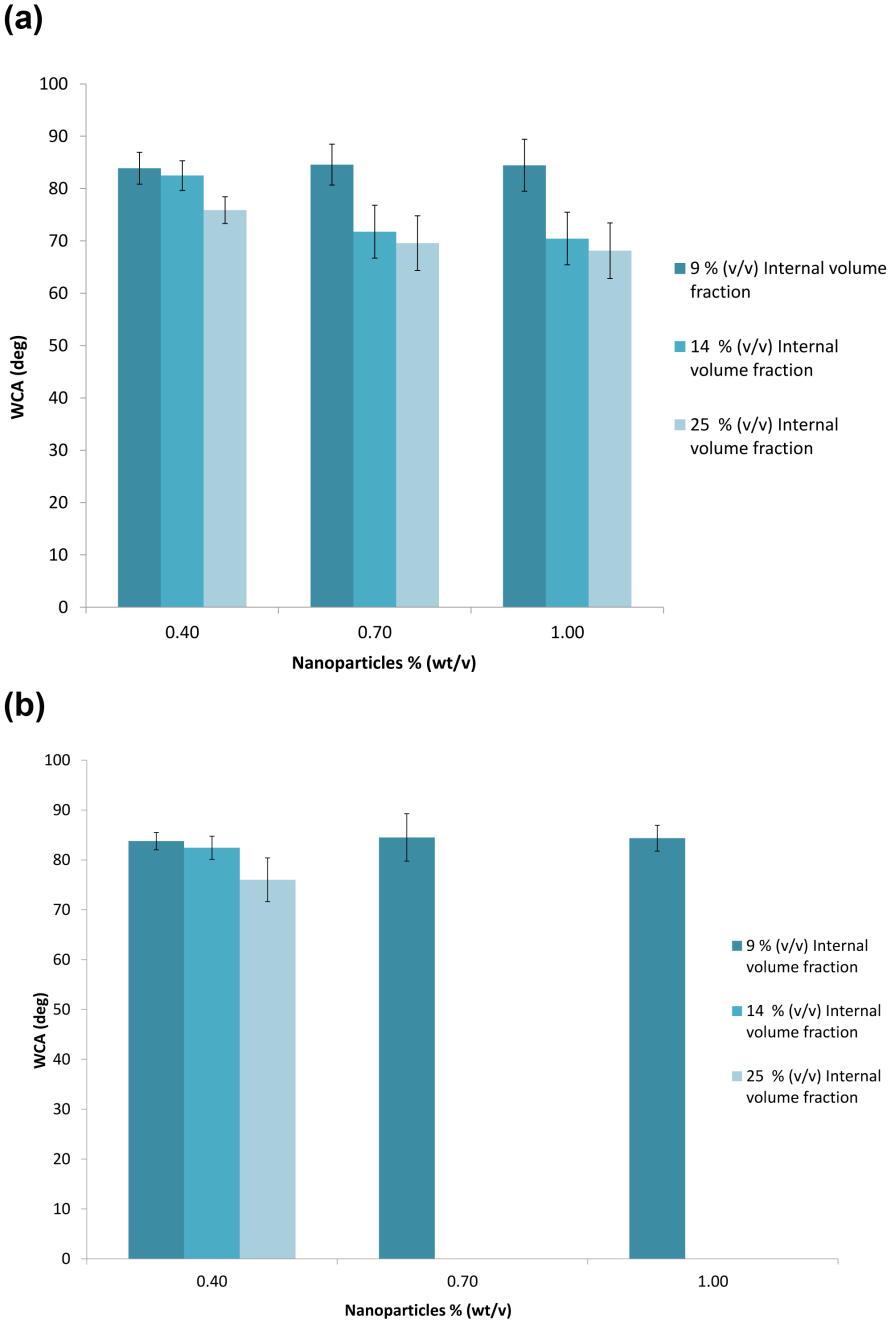
Water contact angle (WCA) measurements on functional membranes with gelation of internal phase. Each series represents an internal volume fraction as displayed in the legend. Horizontal axis represents particle concentration in % (w/w). (**a**) Immediately after deposition of water droplets on the surface, (**b**) 15 seconds after water droplet deposition on the surface. On membranes with 0.7% (w/v) nanoparticle stabilization, the water contact angle decreased to 0 for 14% (v/v) and 25% (v/v) internal volume fractions. Data indicates the average of at least three observations with corresponding standard deviations.

WCAs on the dried functional composite from non-gelling aqueous phase were measured between 75–81° and were stable with time. Composite surfaces were slightly less hydrophobic compared to SBS surfaces due to the presence of salt in the composites. WCAs on dried composites with agarose in the aqueous phase are shown in Figure 7. The samples had nanoparticle concentration of 0.4–1.0% (w/v) and agarose aqueous phase volume fraction Φ of 0.09–0.25. Immediately after addition of water droplets onto the surface, WCAs were measured as 84° for Φ = 0.09 and decreased gradually with increasing Φ (Figure 7a). At Φ = 0.25, WCA was 68°. The initial WCA did not show any dependence on the nanoparticle concentration within 0.4–1.0% (w/v) range. But, at nanoparticle concentration of 0.7% and 1.0%, and at Φ = 0.14 and 0.25, WCA was not stable on the composite surfaces (Figure 7b) and decreased to ∼0° due to penetration of water into the composite film which happened within ∼15 s. This indicates that hydrophilic domains with agarose gel covered most of the top surface of the composite film, probably bicontinuous range of hydrophilic and hydrophobic domains, for Φ = 0.14 and 0.25. These samples showed closed packed functional domains in Figure 5. The water droplets stayed stable on composite surfaces when Φ = 0.09 independent of nanoparticle concentration indicating isolated hydrophilic domains in the hydrophobic SBS matrix (Figure S3). The water droplets also stayed stable on composite surfaces when nanoparticle concentration was 0.4% (w/v) independent of Φ. Smaller nanoparticle concentration can stabilize less interfaces in the emulsion, thus gelled domain size are expected to be larger and the number of gelled domains smaller with increasing Φ. Smaller number of hydrophilic domains will cover less of the top surface of the composite and result in stable water droplets in time.

**Figure 8:**
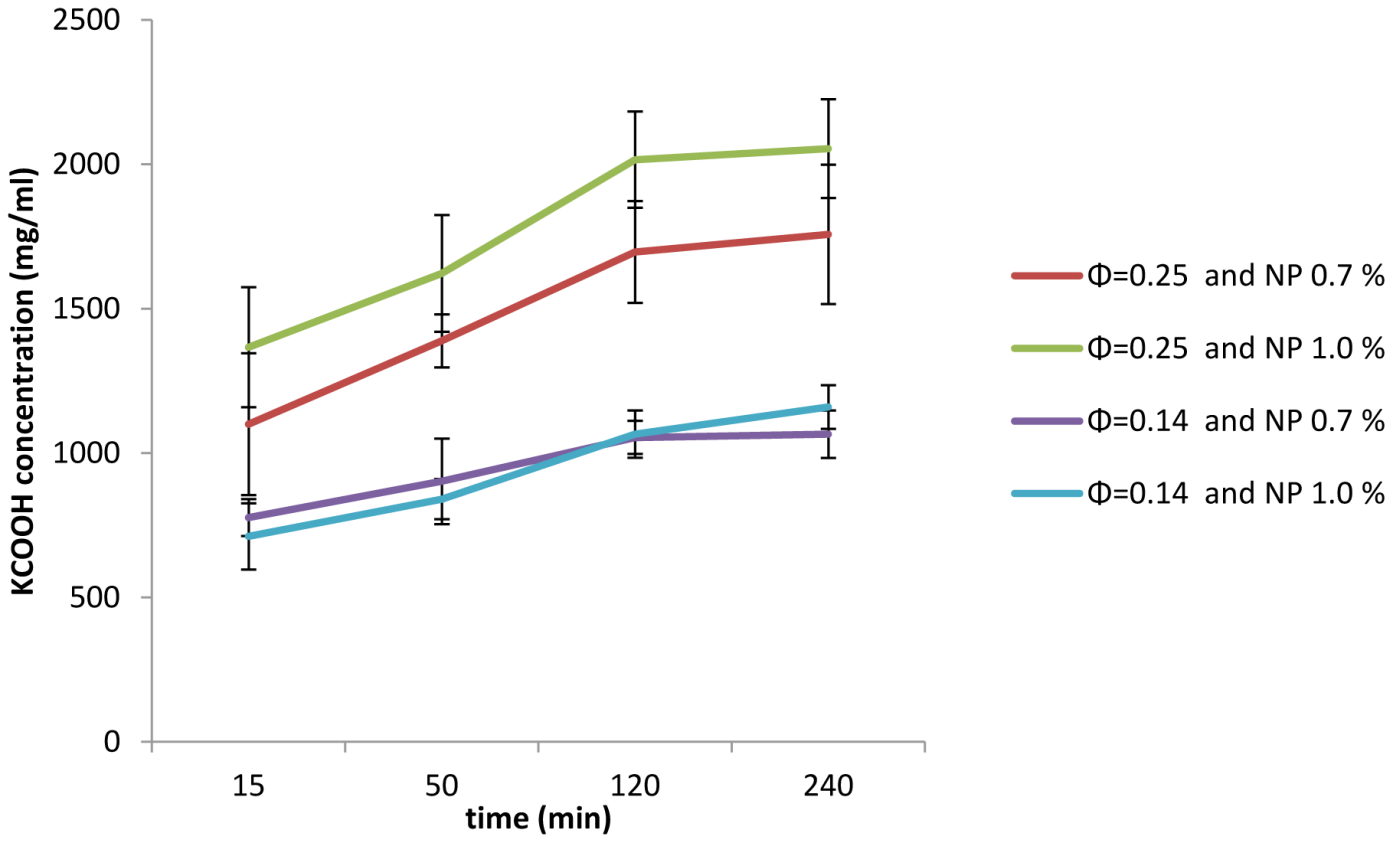
Effect of internal phase volume fraction and nanoparticle concentration on profile of potassium formate release from dry composite membranes with gelation of the internal phase. Data indicates the average of at least three observations with corresponding standard deviations.

Salt release was measured from composites upon incubation in water, where K+ ion concentration was measured at 15, 50, 120 and 240 minutes (Table S2). Figure 8 shows that K+ ion release has an increasing trend from all composites for 2 hours, beyond which the release reaches a plateau up until 4 hours. It has been observed that, the amount of K+ depends on the internal volume fraction and hence initial salt content (Table S3), as a result higher K+ ion were measured for higher internal phase volume fraction. Higher nanoparticle concentration also resulted in an increase in the amount of K+ ion release into the surrounding water medium. It was calculated that the composites with internal phase volume fraction and nanoparticle concentration of (Φ = 0.25, 1.0%), (Φ = 0.25, 0.7%), (Φ = 0.14, 0.7%) and (Φ = 0.14, 1.0%) resulted in the release of 48.9%, 44.4%, 41.8% and 48.3% salt, respectively. Higher K+ ion releases obtained for high nanoparticle concentrations could be explained by smaller sizes and higher number of droplets of internal phase obtained in the composite membrane with high nanoparticle concentrations. Increases in the number and interfacial area of the droplets in the internal phase promoted higher K+ ion release.

**Figure 9:**
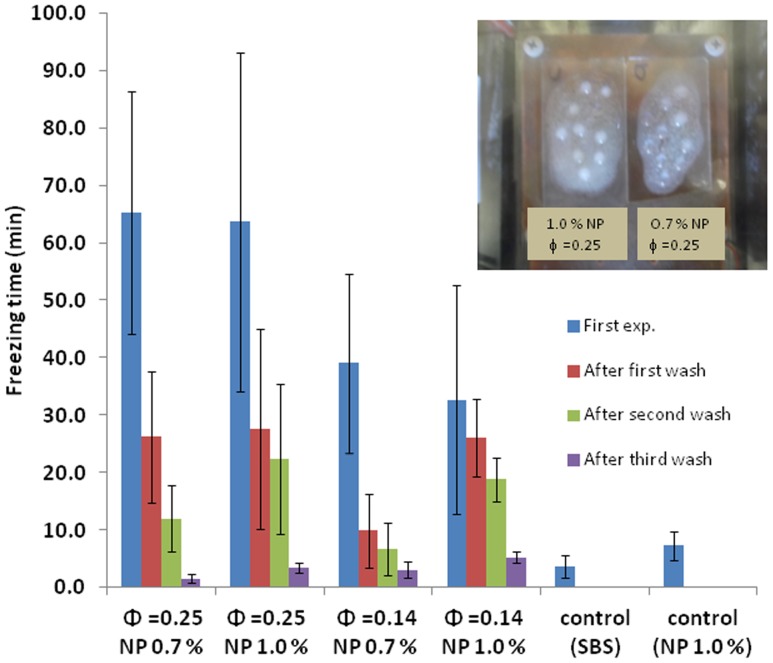
Average freezing times water droplets on dry composite membranes with gelation of the internal phase, SBS (control) and SBS with 1.0% nanoparticle concentration (control). The inset image is taken from temperature and humidity controlled chamber, where the composite with 1% (w/v) nanoparticle concentration (left, Φ = 0.25) and 0.7% (w/v) nanoparticle concentration (right, Φ = 0.25) was monitored at 60 minutes (plate temperature: −14°C, ambient temperature: 5°C). Data indicates the average of at least three observations with corresponding standard deviations.

Hydrophobic SBS films containing homogeneously distributed KCOOH pockets have potential to be used as anti-icing coatings. The release of KCOOH into the water that is in touch with the coating decreases the freezing temperature of water. We have tested the anti-icing properties of the composite films by recording the freezing times of water droplets placed on the composite films. The measurements were done in a temperature and humidity controlled chamber, where the plate temperature was kept at −14°C. The corresponding ambient temperature in the chamber was 5°C which ensured the nucleation of ice on the film surface and allowed us to probe the effectiveness of the composite films. Figure 9 demonstrates freezing times measured on composites after subsequent rinsing steps. The average freezing times were recorded for at least ten water droplets on film surfaces. It can be observed from the Figure that the composites with high internal volume fractions has delayed freezing for about 70 minutes, while a delay of about 40 minutes was recorded with lower internal volume fractions in the composites. This is explained by the presence of high salt concentration included in the high internal phase volume fractions which delayed ice formation and increased freezing time as a result of decreasing the chemical potential of water. Nanoparticles had an opposite effect on freezing time, where composite films prepared with higher nanoparticle concentrations had faster freezing times. Inset in Figure 9 demonstrates the representative image for one of the experiments with composite films in humidity and temperature controlled chamber. It can be observed from the inset in Figure 9 that most of the water droplets on the composite membrane (left, Φ = 0.25) with 1% (w/v) nanoparticle concentration are frozen, while there are still unfrozen droplets on the composite membrane (right, Φ = 0.25) with 0.7% (w/v) nanoparticle concentration after about 60 minutes. Another interesting observation that could be made from these experiments is higher freezing times recorded on membrane surfaces after subsequent washing steps. This could be due to decreased permeability of internal phase to salt diffusion with high nanoparticle concentration, and limited salt release for longer duration. As a result, anti-icing experiments on composite surfaces with subsequent rinsing steps resulted in delayed freezing times.

## Conclusions

The two well established approaches used in the emulsion templating have been studied in this work in order to obtain the hydrophobic membrane that stores the hydrophilic phase therein. Surfactant stabilization method was compared with that of nanoparticles interfacial adsorption and superiority of the particles stabilization technique over the conventional surfactant method has been demonstrated. Further stabilization of the obtained composite materials can be achieved by gelation of the water phase as demonstrated in the comparative experiments. The effect of such parameters as nanoparticles concentration, dispersed phase volume fraction and presence of the gelating agent on the emulsions stability in time, its rheology, the hydrophobicity and morphology as well as the salt release and anti-icing properties of the dry composite membranes, have been investigated in detail.

Apart from using this composite material in form of cast membranes, the designed structures are promising to be functionally loaded with other components and thus can find future applications as monolith scaffolds in tissue engineering, drug delivery, or as a tool in food processing. The designed encapsulation method is applicable to any water soluble substances. Also, the used nanoparticles template method can be applied to prepare emulsions and dry membranes or viscous stable multiphase systems based on other hydrophobic mediums. It might allow the overcoming incompatibilities of used hydrophobic polymers with many water soluble substances.

## Supporting Information

Figure S1 **(a)** Droplet size distribution for emulsions when internal phase volume fraction (Φ) of 0.17, and nanoparticle concentration of 1.0% (w/w) were used. Average droplet size was measured as 120.65 µm, when gelation occurs within the internal phase. Average droplet size was measured as 74.89 µm in the absence of gelation. **(b)** Droplet size distribution for emulsions when internal phase volume fraction (Φ) of 0.33, and nanoparticle concentration of 1.0% (w/w) were used. Average droplet size was measured as 74.3 µm, when gelation occurs within the internal phase. Average droplet size was measured as 55.13 µm in the absence of gelation.
(TIF)

Figure S2 **(a)** Loss and storage moduli in response to frequency for the template emulsion of gel cores (Φ = 0.17, 0.7% wt. particle concentration and 0.17 internal phase fraction). **(b)** Loss and storage moduli in response to frequency for the template emulsion of non-gel cores (0.7% wt. particle concentration and 0.17 internal phase fraction). Data indicates the average of at least three observations with corresponding standard deviations.
(TIF)

Figure S3 **(a)** Scanning electron microscope (SEM) image for the templated dry emulsion with gel cores **(b)** Closer view of the SEM image shown in (a).
(TIF)

Table S1 Average droplet/domain sizes (µm) for all samples in wet and dry states with respect to internal volume fraction (horizontal) and particle concentration % (vertical).
(DOCX)

Table S2 Concentrations of potassium formate in water released from dry composite membranes with gelation in different time periods.
(DOCX)

Table S3 Initial amounts of potassium formate encapsulated in the composites with gelation of the internal phase.
(DOCX)
