# Supplementary figures and images for: Nanoparticle and Gelation Stabilized Functional Composites of an Ionic Salt in a Hydrophobic Polymer Matrix
Source: PLoS One. 2014 Feb 6;9(2):e88125. doi: 10.1371/journal.pone.0088125 (PMC3916421; doi:10.1371/journal.pone.0088125)

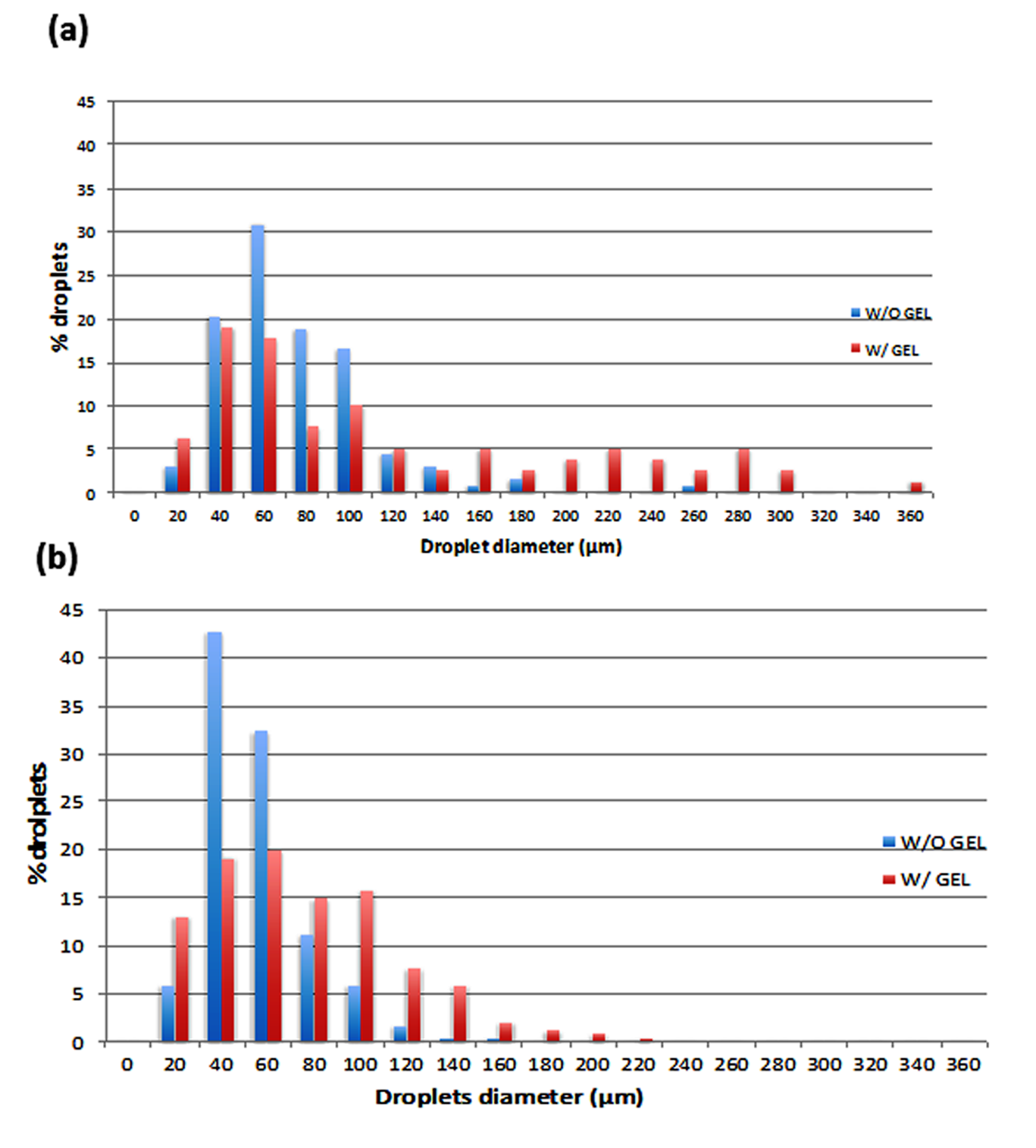

Supplement: Figure S1 — (a) Droplet size distribution for emulsions when internal phase volume fraction (Φ) of 0.17, and nanoparticle concentration of 1.0% (w/w) were used. Average droplet size was measured as 120.65 µm, when gelation occurs within the internal phase. Average droplet size was measured as 74.89 µm in the absence of gelation. (b) Droplet size distribution for emulsions when internal phase volume fraction (Φ) of 0.33, and nanoparticle concentration of 1.0% (w/w) were used. Average droplet size was measured as 74.3 µm, when gelation occurs within the internal phase. Average droplet size was measured as 55.13 µm in the absence of gelation. (TIF) [file pone.0088125.s001.tif]

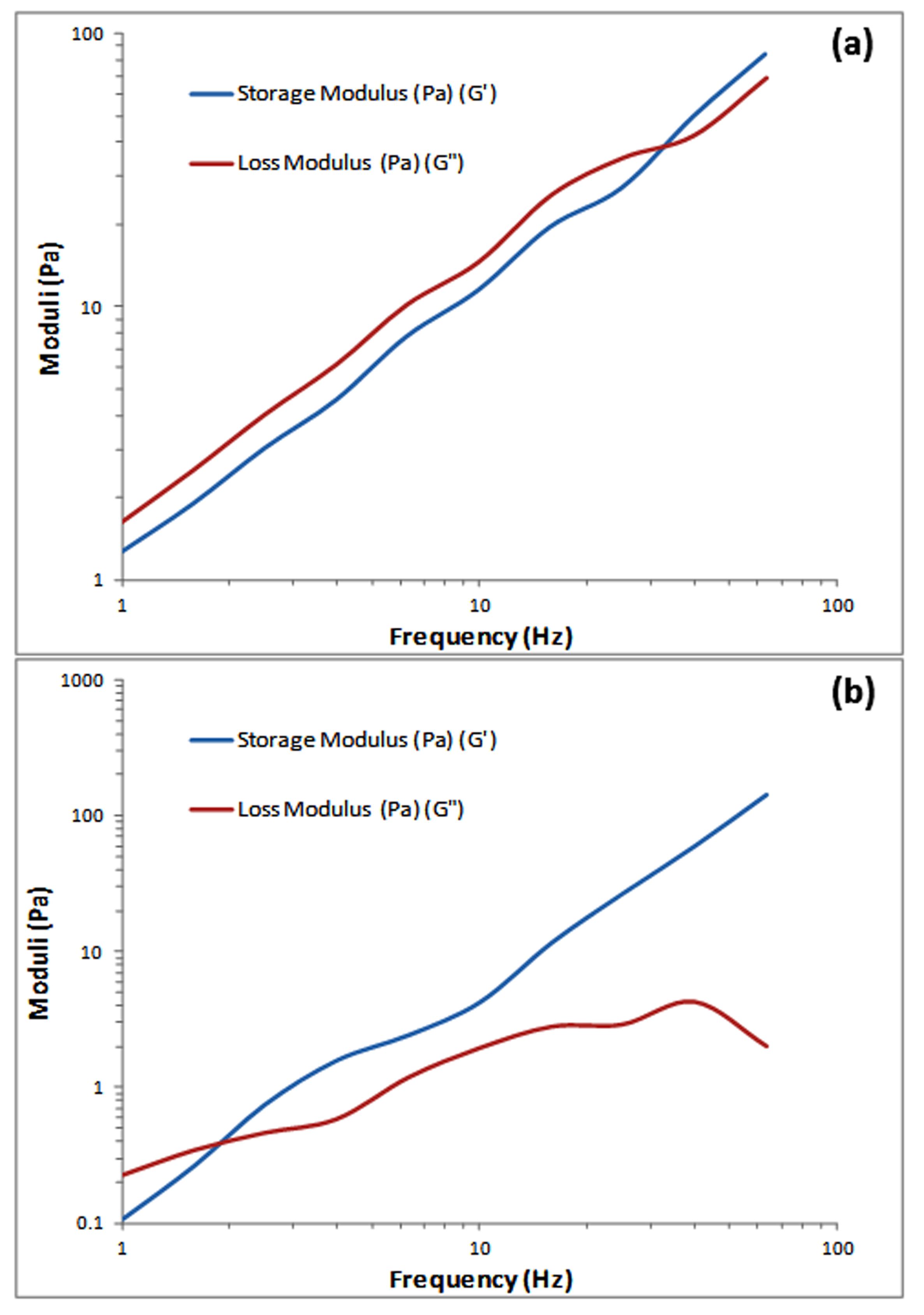

Supplement: Figure S2 — (a) Loss and storage moduli in response to frequency for the template emulsion of gel cores (Φ = 0.17, 0.7% wt. particle concentration and 0.17 internal phase fraction). (b) Loss and storage moduli in response to frequency for the template emulsion of non-gel cores (0.7% wt. particle concentration and 0.17 internal phase fraction). Data indicates the average of at least three observations with corresponding standard deviations. (TIF) [file pone.0088125.s002.tif]

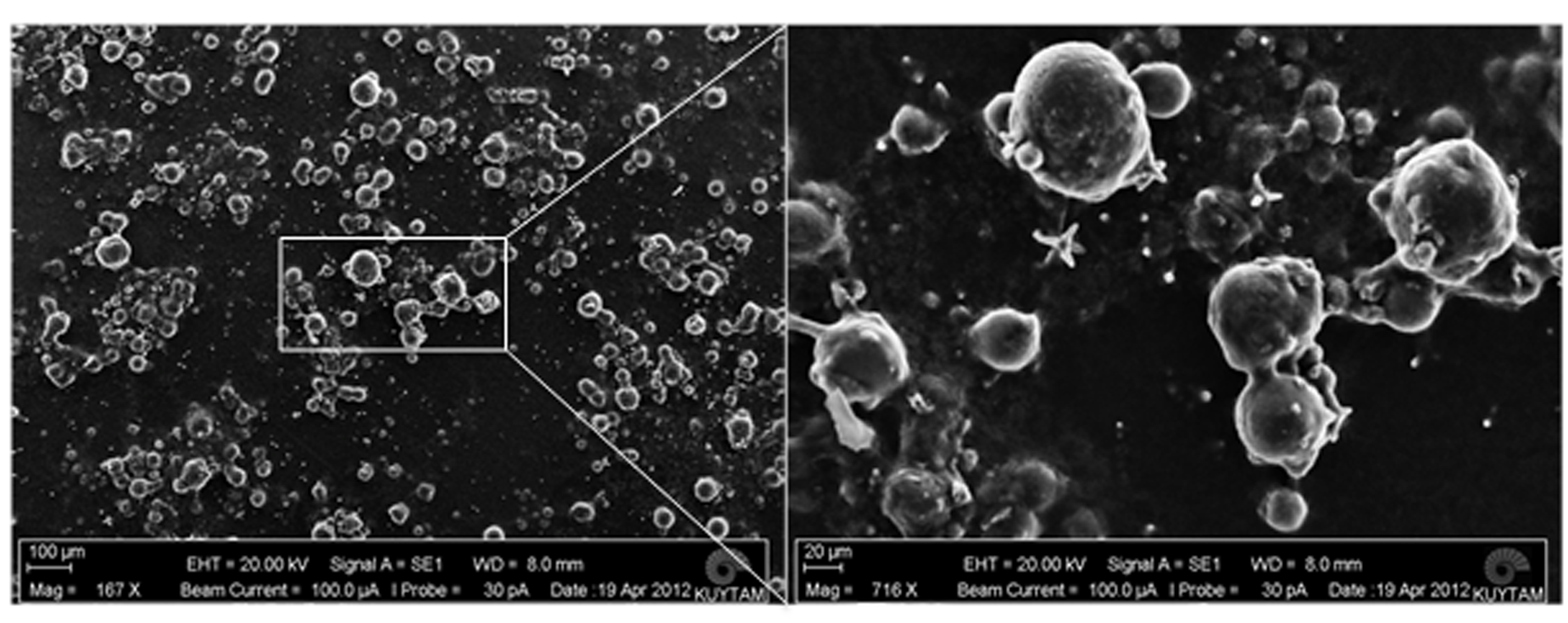

Supplement: Figure S3 — (a) Scanning electron microscope (SEM) image for the templated dry emulsion with gel cores (b) Closer view of the SEM image shown in (a). (TIF) [file pone.0088125.s003.tif]
