# Supplementary material for: Nanoparticle and Gelation Stabilized Functional Composites of an Ionic Salt in a Hydrophobic Polymer Matrix
Source: PLoS One. 2014 Feb 6;9(2):e88125. doi: 10.1371/journal.pone.0088125 (PMC3916421; doi:10.1371/journal.pone.0088125)
Supplement: Table S1 — Average droplet/domain sizes (µm) for all samples in wet and dry states with respect to internal volume fraction (horizontal) and particle concentration % (vertical). (DOCX) [file pone.0088125.s004.docx]

|  | |  | **Nanoparticle concentration % wt (Without gelation in the internal phase)** | | | **Nanoparticle concentration % wt (With gelation in the internal phase)** | | |
| --- | --- | --- | --- | --- | --- | --- | --- | --- |
|  | **Internal volume fraction (%(v/v)** | | **0.4 %** | **0.7%** | **1.0 %** | **0.4 %** | **0.7%** | **1.0 %** |
| **WET** | 9 | | 95.25 | 151.11 | 101.65 | 248.12 | 208.03 | 196.09 |
|  | 14 | | 78.82 | 120.18 | 74.89 | 160.26 | 162.14 | 120.65 |
|  | 25 | | 49.73 | 101.67 | 55.06 | 101.24 | 122.25 | 74.24 |
| **DRY** | 9 | | 91.24 | 120.98 | 95.17 | 240.67 | 175.98 | 175.43 |
|  | 14 | | 78.43 | 112.43 | 73.32 | 158.65 | 166.91 | 119.07 |
|  | 25 | | 41.27 | 96.37 | 49.03 | 102.38 | 89.59 | 73.89 |
