# Supplementary material for: Nanoparticle and Gelation Stabilized Functional Composites of an Ionic Salt in a Hydrophobic Polymer Matrix
Source: PLoS One. 2014 Feb 6;9(2):e88125. doi: 10.1371/journal.pone.0088125 (PMC3916421; doi:10.1371/journal.pone.0088125)
Supplement: Table S2 — Concentrations of potassium formate in water released from dry composite membranes with gelation in different time periods. (DOCX) [file pone.0088125.s005.docx]

| **Time (min)** | **Φ=0.25** | | **Φ=0.14** | |
| --- | --- | --- | --- | --- |
|  | **NP 0.7 % (mg/ml)** | **NP 1.0 % (mg/ml)** | **NP 0.7 % (mg/ml)** | **NP 1.0 % (mg/ml)** |
| **15** | 505 | 627 | 356 | 326 |
| **50** | 637 | 744 | 414 | 386 |
| **120** | 778 | 925 | 484 | 489 |
| **240** | 806 | 943 | 489 | 532 |
