# Supplementary material for: Nanoparticle and Gelation Stabilized Functional Composites of an Ionic Salt in a Hydrophobic Polymer Matrix
Source: PLoS One. 2014 Feb 6;9(2):e88125. doi: 10.1371/journal.pone.0088125 (PMC3916421; doi:10.1371/journal.pone.0088125)
Supplement: Table S3 — Initial amounts of potassium formate encapsulated in the composites with gelation of the internal phase. (DOCX) [file pone.0088125.s006.docx]

|  | **Φ=0.25** | | **Φ=0.14** | |
| --- | --- | --- | --- | --- |
|  | **NP 0.7 % (mg/ml)** | **NP 1.0 % (mg/ml)** | **NP 0.7 % (mg/ml)** | **NP 1.0 % (mg/ml)** |
| **Initial number of moles of KCOOH (mmol)** | 0.99 | 0.99 | 0.56 | 0.56 |
